# Ecuaciones para estimar la talla de ancianos colombianos mediante la altura de la rodilla

**DOI:** 10.7705/biomedica.4820

**Published:** 2019-12-30

**Authors:** María Victoria Benjumea, Alejandro Estrada-Restrepo, Carmen Lucía Curcio

**Affiliations:** 1 Grupo de Investigación Nutral, Universidad CES, Medellín, Colombia Universidad CES Grupo de Investigación Nutral Universidad CES Medellín Colombia; 2 Grupo de Investigación Demografía y Salud, Universidad de Antioquia, Medellín, Colombia Universidad de Antioquia Grupo de Investigación Demografía y Salud Universidad de Antioquia Medellín Colombia; 3 Grupo de Investigación en Gerontología y Geriatría, Universidad de Caldas, Manizales, Colombia Universidad de Caldas Grupo de Investigación en Gerontología y Geriatría Universidad de Caldas Manizales Colombia

**Keywords:** predicción, estatura, anciano, estado nutricional, encuestas epidemiológicas, Colombia, Forecasting, body height, aged, nutritional status, health surveys, Colombia

## Abstract

**Introducción.:**

La estatura en el anciano no refleja su talla real de adulto joven debido al envejecimiento de su columna vertebral, entre otros aspectos.

**Objetivo.:**

Proponer ecuaciones para estimar la talla de los ancianos colombianos mediante la altura de la rodilla, según el grupo étnico y el sexo.

**Materiales y métodos.:**

Se hizo un análisis secundario del estudio transversal SABE 2015, utilizando un diseño muestral probabilístico y multietápico en personas colombianas de 60 o más años. Se seleccionaron aleatoriamente dos grupos de la base de datos del estudio SABE: el grupo para el desarrollo de las ecuaciones y el grupo para su validación. Se hizo un análisis de regresión lineal múltiple para estimar la estatura mediante la altura de la rodilla en los grupos étnicos (indígenas, afrodescendientes y blancos-mestizos) por edad y sexo; los resultados se validaron en cada subgrupo de estudio.

**Resultados.:**

Se diseñaron seis ecuaciones por sexo (hombres=3.665, mujeres=3.019) y etnia; los coeficientes de determinación ajustados (R2 ) de las ecuaciones en hombres de los tres grupos étnicos oscilaron entre 64 y 75 % y, los errores estándar, entre 3,09 y 3,93 cm. En las mujeres, los R2 de las tres ecuaciones fluctuaron entre 53 y 73 % y los EE, entre 2,96 y 3,90 cm.

**Conclusión.:**

La ecuación con mejor capacidad para estimar la talla del anciano colombiano fue la obtenida para los afrodescendientes de ambos sexos, en tanto que en la población indígena se presentaron los menores coeficientes de determinación.

## Abstract (en)

**Introduction:**

The height in the elderly does not reflect their real size as young adults due to the aging of their spine, among other aspects.

**Objective:**

To estimate the equations to predict the height in Colombian elders according to their ethnic group and sex using the knee height measurement.

**Materials and methods:**

We conducted a secondary analysis of the SABE, 2015, crosssectional study using a multistage probabilistic sampling design in people aged 60 years and over in Colombia. We randomly selected two groups from the study’s database: A development group and a validation group of the equations. Age and anthropometric characteristics were similar in both groups. We performed a multiple linear regression analysis to predict the height using knee height measurement in the different ethnic groups (Indigenous, Afro-descendant, and white-mestizo groups) by age and sex; the results were validated in each selected subgroup.

**Results:**

We designed six equations by sex (men=3,665; women=3,019) and ethnic group. The adjusted R2 of the equations in men from the three ethnic groups oscillated between 64% and 75% and the standard errors, between 3,09 and 3,93 cm while in women, the R2 s of the three equations ranged between 53% and 73% and the EEs, between 2,96 and 3,90 cm.

**Conclusion:**

The equation with the best predictive capacity of the height of Colombian elders was obtained for African descendants of both sexes. The lowest coefficients of determination were obtained for the indigenous population.

La estatura de los ancianos no refleja su talla real de adultos jóvenes debido al envejecimiento de la columna vertebral, entre otros aspectos ^1^^,^^2^. Por lo tanto, en este grupo etario el índice de masa corporal (IMC), empleado en antropometría para evaluar el estado nutricional, no debe calcularse con la talla real del anciano, pues así se subestima la desnutrición, condición que es más riesgosa para la vida de los adultos mayores que el exceso de peso. Esta práctica también conduce a una mala interpretación de su estado nutricional y a adoptar conductas equivocadas en la atención de su salud y su nutrición ^3^^,^^4^.

La talla del anciano debe estimarse con segmentos corporales que hayan soportado el estrés nutricional durante sus períodos críticos de crecimiento y que, además, reflejen al máximo la talla que pudo haber tenido en su vida adulta joven ^5^^-^^7^.

A partir de la información disponible en las encuestas de salud, bienestar y envejecimiento (SABE) de varios países, se han diseñado ecuaciones de estimación de la talla del anciano con la altura de la rodilla como variable dependiente ^8^. Entre dichos estudios, se destacan los de Jamaica, Brasil, Chile y Ecuador ^9^^,^^10^. 

Algunos autores han utilizado otros segmentos corporales para aproximarse a la talla real del anciano con medidas como la 'braza' y la altura de la rodilla hasta el talón ^11^^-^^13^; según los resultados publicados, la altura de la rodilla es el segmento corporal que mejor permite estimar la talla que el adulto mayor tuvo en su juventud ^14^. 

En Colombia, no se han diseñado ecuaciones para la evaluación del estado nutricional de este grupo poblacional, por lo que se han tenido que emplear las de otros países con etnias y condiciones diferentes, por ejemplo, las publicadas por Chumlea, et al. ^2^^,^^15^^,^^16^. 

En este contexto, es necesario analizar los datos de la talla y la altura de la rodilla disponibles en la encuesta SABE del 2015 ^17^, con el fin de obtener ecuaciones de estimación de la talla de los ancianos colombianos en su juventud considerando su etnia, edad y sexo para evaluar apropiadamente su estado nutricional a partir del IMC.

## Materiales y métodos

El estudio se llevó a cabo utilizando los datos de la encuesta de salud, bienestar y envejecimiento (SABE) de Colombia para el 2015. Fue un estudio observacional y transversal en el cual se evaluaron personas de 60 o más años, que no estaban ingresadas en instituciones y vivían en áreas urbanas y rurales de Colombia.

Los sujetos fueron seleccionados mediante un muestreo por conglomerados, multietápico, probabilístico y estratificado. La muestra evaluada se captó en todos los departamentos del país e incluyó a 23.694 adultos mayores representativos de las grandes ciudades, las regiones y del país. 

La base de datos de la SABE fue suministrada por el Ministerio de Salud y Protección Social de Colombia; en ella, la información se maneja de forma anónima según las disposiciones pertinentes ^17^. Desde el inicio del estudio SABE, se excluyeron los sujetos que presentaron un puntaje de menos de 13 en la versión revisada del Mini-Mental State Examination (MMSE); además, en este estudio, se excluyeron también a aquellos que no registraban datos en las variables de estudio o que presentaban valores biológicamente imposibles en la talla o en la altura de la rodilla.

La información detallada sobre los aspectos metodológicos del estudio SABE 2015, se puede consultar en la publicación de Gómez, et al. ^17^, o en el enlace https://www.minsalud.gov.co/sites/rid/Lists/BibliotecaDigital/RIDE/ VS/ED/GCFI/doc-metodologia-sabe.pdf

### Variables analizadas

Las mediciones antropométricas fueron tomadas por personal entrenado especialmente para la encuesta SABE del 2015. Las variables antropométricas consideradas en el presente estudio fueron la talla y la altura de la rodilla, las cuales se midieron utilizando las técnicas propuestas por Lohman, *et al*. ^18^. Las mediciones para ambas variables se tomaron dos veces y, si la diferencia entre ambas excedía los 0,5 cm para la talla y los 0,5 cm para la altura de la rodilla, se hacía una tercera medición para promediar las dos mediciones más cercanas. Se incluyeron, además, variables demográficas como el sexo, la edad y la etnia declarada por los mismos participantes.

### Análisis estadístico

A partir de la base de datos de la encuesta SABE 2015, se generaron dos subgrupos de forma aleatoria: el primero, para el diseño de las ecuaciones y, el segundo, para su validación.

Mediante estadística descriptiva, se caracterizó a los adultos mayores de ambos grupos. Las variables numéricas se expresaron en rangos, promedios, medianas y desviación estándar, y las demás variables, en frecuencias absolutas y relativas. Después de comprobar el supuesto de normalidad mediante la prueba de Kolmogorov-Smirnov, se compararon las variables de edad en años y de talla y altura de la rodilla en centímetros entre los subgrupos con la prueba t de Student para grupos independientes o, en su defecto, la prueba U de Mann Whitney.

En el diseño de las ecuaciones, se utilizó un análisis de regresión lineal múltiple en el subgrupo, con cuyos datos se diseñaron las ecuaciones tomando la talla como variable dependiente y, la edad y la altura de la rodilla, como variables independientes.

Las ecuaciones se diseñaron para población indígena, afrodescendiente y blanco-mestiza por sexo. Cada uno de los modelos diseñados buscó ajustarse a los supuestos de linealidad, normalidad, homocedasticidad y colinealidad de residuos. El coeficiente de determinación (R^2^ ) ajustado y el error estándar (EE) de la estimación se utilizaron para evaluar la precisión de las ecuaciones.

Los modelos de estimación determinados con los datos del grupo de diseño se aplicaron en el subgrupo de validación según lo exigido para este tipo de análisis. El R^2^ ajustado y el error puro se calcularon para valorar la precisión de las ecuaciones. Así, un R^2^ elevado y un error puro bajo indicaban una mayor precisión de la ecuación. Todos los análisis estadísticos se hicieron en el programa R ^19^. La significación estadística se estableció como p<0,05.

### Consideraciones éticas

El estudio SABE recibió aprobación ética de los comités de la Universidad de Caldas (Acta No. CBCS-021-14) y de la Universidad del Valle (Actas No. 09-014 y O11-015). Los adultos mayores suministraron el consentimiento informado por escrito y su participación fue voluntaria. En todas las etapas del estudio, se mantuvo la confidencialidad de los datos suministrados por los encuestados. El estudio fue desarrollado de acuerdo con los principios de la Declaración de Helsinki y de la Resolución 008430 de 1993 del Ministerio de Salud de Colombia ^20^. El estudio no representó riesgo para la población estudiada.

## Resultados

Del total de ancianos estudiados (n=11.922), el 44,1 % era de sexo masculino. Más de la mitad (74,1 %) vivía en la zona urbana de Colombia. Con respecto a la etnia, el 13,7 % se declaró como indígena, el 12,2 %, como afrodescendiente, y el 72,7 %, como blancos-mestizos; la edad mínima fue de 60 años y la máxima de 99 años, con un promedio de 69,2+7,1 años.

La edad y las características físicas de cada uno de los subgrupos conformados se presentan en el cuadro 1. No se encontraron diferencias significativas en los promedios o medianas (p>0,05) de la edad, la talla o la altura de la rodilla, entre los subgrupos de cada una de las etnias, ni por sexo (cuadro 1).

**Cuadro 1:** Comportamiento de las variables de interés por subgrupos

				Subgrupo de construcción					Subgrupo de validación								
	Variable			de ecuaciones													p*
												
			n	Rango	X±S	Mediana		n	Rango	X±S				Mediana		
															
	Indígenas hombres														
	Edad (años)	569		60-93	68,8±6,8	67,0	280		60-89	68,7±6,7		67,0			0,789
	Talla (cm)	528		145,0-181,4	162,0±6,6	162,0	260		144,0-180,0	162,2±6,7		162,0			0,67**
	Altura de rodilla (cm)	554		39,4- 59,2	50,2±2,9	50,2	271		42,0- 57,0	50,3±2,7		50,4			0,412
	Indígenas mujeres														
	Edad (años)	519		60-91	68,2±6,7	66,0	270		60-86	68,6±6,7		67,0			0,313
	Talla (cm)	466		136,0-169,5	149,8±5,8	149,2	241		135,0-165,0	150,4±6,4		150,0			0,217
	Altura de rodilla (cm)	501		35,3- 54,5	46,2±2,9	46,2	259		38,7- 55,0	46,2±3,0		46,3			0,856
	Afrodescendientes hombres														
	Edad (años)	457		60-95	69,2±7,3	68,0	248		60-91	68,8±6,8		68,0			0,636
	Talla (cm)	416		149,5-182,2	165,8±6,2	166,0	223		149,0-184,0	166,2±6,6		166,5			0,43**
	Altura de rodilla (cm)	451		43,3- 60,2	51,8±2,9	51,9	238		44,3- 59,8	52,0±2,8		52,1			0,54**
	Afrodescendientes mujeres														
	Edad (años)	495		60-91	68,1±6,8	66,0	256		60-90	68,3±6,7		67,0			0,683
	Talla (cm)	452		143,0-174,0	155,1±5,9	154,4	233		142,0-174,0	154,2±6,2		153,8			0,05**
	Altura de rodilla (cm)	485		38,0- 57,0	48,4±2,9	48,2	249		39,5- 59,0	48,3±2,9		48,4			0,789
	Blancos-mestizos hombres														
	Edad (años)	2.312		60-99	69,6±7,2	68,0	1.311		60-98	69,9±7,3		69,0			0,176
	Talla (cm)	2.111		146,0-179,0	163,2±5,8	163,3	1.193		145,5-185,0	163,5±6,6		163,0			0,712
	Altura de rodilla	2.195		39,9- 59,0	50,6±2,5	50,7	1.246		40,2- 59,0	50,6±2,8		50,5			0,918
	Blancos-mestizos mujeres														
	Edad (años)	3.228		60-97	69,4±7,2	68,0	1.812		60-95	69,2±7,2		68,0			0,233
	Talla (cm)	2.813		137,0-170,0	150,8±5,1	150,5	1.604		138,0-170,0	151,2±5,7		151,0			0,069
	Altura de rodilla (cm)	3.047		32,1- 58,0	46,5±2,7	46,5	1.731		37,0- 56,1	46,6±2,7		46,6			0,180

* Prueba U de Mann Whitney, a menos que se indique otra cosa.

** Prueba t de Student para grupos independientesientes

X±S: promedio ± desviación estándar

Las ecuaciones de estimación de la talla de los ancianos colombianos diseñadas para cada sexo y etnia, se presentan en el cuadro 2. Los R2 de los modelos oscilaron entre 0,53 (mujeres indígenas) y 0,75 (hombres afrodescendientes). Los errores de estimación de los modelos fueron menores de 3,94 cm.

**Cuadro 2:** Modelos de predicción de la talla del anciano por etnia y sexo

Modelos*	n	_R_ **2**	EE	Normalidad	Homocedasticidad
Indígenas hombres:					
Talla = 82,695 + 1,745 (AR) - 0,121 (edad)	522	0,64	3,9310	0,9194	0,1060
Indígenas mujeres:					
Talla = 90,281 + 1,436 (AR) - 0,102 (edad)	455	0,53	3,9010	0,3649	0,3055
Afrodescendientes hombres:					
Talla = 79,298 + 1,855 (AR) - 0,141 (edad)	414	0,75	3,0850	0,4557	0,5583
Afrodescendientes mujeres:					
Talla = 76,233 + 1,767 (AR) - 0,098 (edad)	443	0,73	3,1070	0,4930	0,8819
Blancos-mestizos hombres:					
Talla = 75,514 + 1,883 (AR) - 0,108 (edad)	2.083	0,69	3,2620	0,0902	0,0744
Blancos-mestizos mujeres:					
Talla = 86,497 + 1,553 (AR) - 0,119 (edad)	2.767	0,66	2,9590	0,0900	0,6466

* Todos los modelos presentaron valores de inflación de la varianza inferiores a 5.

AR: altura de rodilla en cm; talla en cm; edad en años; R2: coeficiente de determinación; EE: error estándar de la estimación

Los diferentes modelos de estimación de la talla se aplicaron en el subgrupo de validación para cada etnia y sexo. Los R2 de las diferentes ecuaciones en el proceso de validación oscilaron entre 0,542 y 0,697. Los errores puros de los modelos estuvieron entre 2,51 y 5,07 (cuadro 3).

**Cuadro 3:** Validación de las ecuaciones para estimar la talla en el grupo de validación

Modelos	n	R^2^	Error puro
Indígenas hombres	255	0,6086	5,0695
Indígenas mujeres	234	0,5420	3,8601
Afrodescendientes hombres	215	0,6535	2,8636
Afrodescendientes mujeres	229	0,6970	2,5100
Blancos-mestizos hombres	1.173	0,6301	3,7550
Blancos-mestizos mujeres	1.589	0,5679	3,5071

R2 : coeficiente de determinación

## Discusión

Además de la disminución de la talla debido al envejecimiento ^21^^,^^22^, las diferencias encontradas en la talla de los ancianos colombianos en este estudio justificaban el diseño de ecuaciones de estimación de esta importante medida antropométrica para cada grupo étnico, tal como lo han planteado los investigadores que han diseñado ecuaciones de estimación de la talla en otros contextos ^1^^,^^22^^,^^23^. El dimorfismo sexual también respalda el ajuste de las ecuaciones de predicción de la talla por sexo, pues las mujeres son más bajas en estatura que los hombres ^14^, lo que se comprobó en los ancianos estudiados.

La talla es una medida esencial en la evaluación nutricional de un individuo. De hecho, la tamización de la desnutrición mediante, por ejemplo, el cálculo del índice de masa corporal, la estimación de la composición corporal mediante el análisis de la bioimpedancia, o la predicción de las necesidades energéticas con las ecuaciones de Harris-Benedict recurre en gran medida a esta medida antropométrica. Además, la talla es necesaria para otros propósitos importantes, como la adaptación de las dosis de los medicamentos citostáticos mediante el cálculo de la superficie total corporal. Infortunadamente, en algunos entornos (por ejemplo, en las unidades de cuidados intensivos) y en condiciones de confinamiento en cama, la evaluación correcta del estado nutricional mediante procedimientos estándar es obviamente inaplicable. Ante este problema, y dada la inexactitud de la medición de la longitud supina, se han estudiado diferentes indicadores indirectos (por ejemplo, la altura de la rodilla o la longitud del brazo) para obtener fórmulas de predicción de la talla ^24^.

Otros elementos que sustentan el diseño de ecuaciones de estimación para la talla real del anciano entrañan problemas, por ejemplo, si se usa la talla del anciano para calcular el índice de masa corporal, se puede subestimar el déficit de peso ^22^^,^^25^ que, como bien se ha descrito, representa un mayor riesgo de mortalidad para el anciano que el exceso de peso ^26^. Por tal razón, es pertinente el uso de otras medidas antropométricas en los ancianos o en individuos en cama para estimar su talla real.

Desde el punto de vista biológico, el uso de la altura de la rodilla en las seis ecuaciones de predicción de la talla de los ancianos colombianos en este estudio se justifica por ser un componente directo de la talla, dada su correlación estrecha con ella ^1^^,^^2^ y, además, por su estabilidad a lo largo de la vida al no presentar cambios sustanciales en momentos de estrés nutricional durante el crecimiento lineal ^14^. Aunque en algunos estudios se plantea que la 'braza' es una medida antropométrica más precisa que la altura de la rodilla para estimar la talla real ^22^^,^^27^, la dificultad para obtenerla, dados los cambios morfopatológicos de los ancianos o de los pacientes en cama, le resta validez y reproducibilidad en el ámbito clínico ^14^.

Entre los grupos de investigación que más han usado la altura de la rodilla como predictor de la talla, se encuentra el de Chumlea, *et al*. ^2^, quienes desde 1985 vienen publicando sus hallazgos sobre la estimación de la talla en ancianos discriminando por sexo, edad y etnia las ecuaciones de regresión obtenidas en poblaciones de diferente tamaño ^1^^,^^15^^,^^16^. En los demás estudios ^(5, 23,28-30)^, con excepción de los que han desarrollado las ecuaciones a partir de los datos de las encuestas SABE de otros países y del estudio de Costa Rica ^31^, los tamaños de la población de estudio no han sido suficientes para diseñar ecuaciones que empleen la altura de la rodilla.

Al comparar este estudio con algunos de los mencionados, se pudo observar que los errores estándar de las ecuaciones propuestas para los ancianos colombianos fueron iguales, o incluso más bajos, en algunas etnias. En el caso del estudio de Chumlea, et al., en 1998 ^16^, los errores oscilaron entre 3,25 y 4,11 en hombres y entre 3,45 y 4,18 en mujeres; igual sucedió con el estudio de Mendoza, *et al*. ^32^, en el que los errores fueron más altos que en el nuestro. Lo anterior ratifica lo importante que resulta el diseño de ecuaciones específicas para nuestra población y el poder contar con una muestra aleatoria de validación independiente y con características similares a la muestra empleada para el diseño de dichas ecuaciones, tal como se logró en este estudio.

El presente estudio pone a disposición de la comunidad académica y científica del país seis ecuaciones de estimación de la talla para ser validadas en ancianos de distintos grupos étnicos con miras a cualificar la evaluación del estado nutricional del anciano en Colombia. Sin embargo, el uso de las fórmulas diseñadas en cada etnia para la estimación de la estatura debe hacerse con precaución, especialmente cuando se haga en sujetos con valores de estatura, altura de la rodilla y edad por debajo de los mínimos o por encima de los máximos con que fueron calculadas las ecuaciones de este estudio.

El estudio presenta algunas fortalezas y limitaciones. Entre las fortalezas se destaca el haber desarrollado ecuaciones con variables sencillas de medir en la población adulta mayor, que incluso puede estar en cama o con dificultades para adoptar posturas rectas y en bipedestación; asimismo, pueden usarse en ámbitos institucionales o domiciliarios. Otra fortaleza es que el diseño de las ecuaciones utilizando una base de datos de población colombiana, permitió el análisis específico por etnia y, además, se contó con un rango amplio de edad y condiciones socioeconómicas. Esto último resulta de especial interés, dadas las desigualdades económicas y sociales de la población colombiana.

Una de las limitaciones fue el menor tamaño de la muestra en la población indígena, comparado con el de las otras etnias, lo que explicaría que fuera este grupo poblacional el que presentó los menores coeficientes de determinación. Lo anterior se debe a que la SABE no tuvo en cuenta la etnia en el muestreo de la población de estudio.
